# Genotyping Approach for Potential Common Source of *Enterocytozoon bieneusi* Infection in Hematology Unit

**DOI:** 10.3201/eid2509.190311

**Published:** 2019-09

**Authors:** Guillaume Desoubeaux, Céline Nourrisson, Maxime Moniot, Marie-Alix De Kyvon, Virginie Bonnin, Marjan Ertault De La Bretonniére, Virginie Morange, Éric Bailly, Adrien Lemaignen, Florent Morio, Philippe Poirier

**Affiliations:** Université de Tours, Tours, France (G. Desoubeaux);; CHU Bretonneau, Tours (G. Desoubeaux, M.-A. De Kyvon, M. Ertault De La Bretonniére, V. Morange, É. Bailly, A. Lemaignen);; Université Clermont-Auvergne, Aubière, France (C. Nourrisson, M. Moniot, V. Bonnin, P. Poirier);; CHU Gabriel-Montpied, Clermont-Ferrand, France (C. Nourrisson, M. Moniot, P. Poirier);; CHU Hôtel Dieu, Nantes, France (F. Morio); Université de Nantes, Nantes (F. Morio)

**Keywords:** microsporidia, microsporidiosis, *Enterocytozoon bieneusi*, genotyping, cluster, ITS rDNA, satellites, epidemiology, outbreak, fungi, France

## Abstract

Microsporidiosis is a fungal infection that generally causes digestive disorders, especially in immunocompromised hosts. Over a 4-day period in January 2018, 3 patients with hematologic malignancies who were admitted to the hematology unit of a hospital in France received diagnoses of *Enterocytozoon bieneusi* microsporidiosis. This unusually high incidence was investigated by sequence analysis at the internal transcribed spacer rDNA locus and then by 3 microsatellites and 1 minisatellite for multilocus genotyping. The 3 isolates had many sequence similarities and belonged to a new genotype closely related to genotype C. In addition, multilocus genotyping showed high genetic distances with all the other strains collected from epidemiologically unrelated persons; none of these strains belonged to the new genotype. These data confirm the epidemiologic link among the 3 patients and support a common source of infection.

Microsporidia are spore-forming eukaryotic and opportunistic intracellular pathogens related to fungi (*1*,*2*). Microsporidiosis usually occurs in the form of isolated cases in immunocompromised patients, including HIV-infected persons and solid-organ transplant recipients (*1*) but can also arise in travelers and is common in children in developing countries. Infection causes digestive disorders, including diarrhea (*1*,*3*). Microsporidia are orally transmitted by interindividual contacts and likely less frequently transmitted by foodborne or waterborne spores from excreta of a wide range of host species (*1*,*4*). At least 16 microsporidian species have been described in humans, but *Enterocytozoon bieneusi* is the most common (*5*). However, little is known about the actual epidemiology of *E. bieneusi* microsporidiosis, and there is a need for a better understanding of its pathophysiology and parasitic cycle (*3*,*5*). Unfortunately, epidemiologic studies are complicated because *E. bieneusi* infection has a low incidence rate worldwide (*6*), and its microbiological diagnosis is difficult and likely often overlooked (*7*). In addition, the species is not easy to cultivate in vitro in routine practice. Investigations can be carried out directly only from infected biologic samples, which usually use DNA from fecal specimens (*1*,*3*,*7*).

More than 250 genotypes of *E. bieneusi* have been identified on the basis of their internal transcribed spacer (ITS) region (*8*,*9*). Depending on the ITS genotypes, zoonotic or host-adapted groups have been identified within species. Phylogenetic studies were able to distinguish >10 groups; most ITS genotypes belonged to group 1, which contains genotypes found in both humans and animals, including cats, pigs, and cattle (*2*). However, ITS sequencing has certain limitations because the same ITS genotype can be isolated from different host species and from different regions. This possible strain diversity within 1 ITS genotype cannot be addressed by a single sequence-based genotyping technique (*10*).

In human and animal medicine, several molecular techniques have been developed to investigate the epidemiology of transmissible agents (*11*–*15*), such as multilocus sequence typing (MLST) analysis or mini/microsatellite length polymorphism using short tandem-repeat markers. A MLST method was developed in 2011 to discriminate among *E. bieneusi* isolates (*16*). Four loci were analyzed with 1 minisatellite (MS4) and 3 microsatellites (MS1, MS3, and MS7). The combination of these 4 markers with the ITS genotype allows for the determination of multilocus genotypes (MLGs). MLG analyses are useful to discriminate between isolates derived from various hosts (*17*) and to detect mixed infections (*18*).

To assess epidemiologic links among 3 cases of *E. bieneusi* infections occurring concomitantly in a single hematology unit in a hospital in France, we used the MLG analysis method. The results showed the utility of this approach in the investigation of a cluster of cases.

## Materials and Methods

### Ethics

The study patients gave informed consent for the use of their samples in the research project. All their personal data were anonymous. We received approval from the ethics committee of the University Hospital of Tours (Center 1, Espace de Réflexion Éthique, Région Centre-Val de Loire, France). The study registration number 2015_003 was issued by the National Commission for Information Technology and Individual Freedom (Commission Nationale de l’Informatique et des Libertés) on January 10, 2015. We performed the study in accordance with the Code of Ethics of the World Medical Association (Declaration of Helsinki) and complied with BRISQ guidelines (*19*). We received technical and financial support from the French Microsporidiosis Network, assisted by the Clinical Research and Innovation Department (DRCI) of the University Hospital of Clermont-Ferrand (Center 2) and the French National Reference Center for Cryptosporidiosis.

### Context of the Study

Center 1 is a university hospital in France that contains 2,008 inpatient beds and comprises 3 main sites spread over a distance of a few kilometers. The hematology unit is located in the Center for Adult Medicine (Tours, France; latitude 47.3900474, longitude 0.6889268). Systematic surveillance and exhaustive registration of all cases of microsporidiosis began in the hospital in January 2011. During January 2011–January 2018, there were 33,769 inpatient admissions. Only 2 cases of *E. bieneusi* were diagnosed in the hematology unit and the oncology department in patients admitted in 2017; these cases occurred at different times. In contrast, 3 patients with hematologic malignancies, designated M01-05 to M01-07, received diagnoses of *E. bieneusi* microsporidiosis during a 4-day period, January 12–16, 2018.

### Biologic Procedures for Routine Diagnosis of *E. bieneusi* Infection

All diarrheic fecal specimens and feces obtained from high-risk patients, including HIV-positive persons, solid-organ/bone-marrow transplant recipients, and patients with cancer or autoimmune diseases (*1*,*20*), were systematically screened for microsporidia in the parasitology–mycology laboratory of center 1. The first-line diagnostic procedure used a real-time qualitative PCR (qPCR) that targets the 18S rRNA gene of *E. bieneusi*. We performed genomic DNA extraction with the QIAmp DNA Stool Mini Kit (QIAGEN, https://www.qiagen.com), according to the manufacturer’s instructions. We stored DNA extracts at −20°C until subsequent analysis. We completed amplification with Eb and Eb5 oligonucleotide primers at a final concentration of 0.5 μmol/L and detected the 180-bp product using the specific fluorescent TaqMan probe EbS2 in the LightCycler 480 II apparatus (Roche, https://www.roche.com) as described previously (*7*,*21*). We set the positive cutoff value of qPCR at <39 quantitative cycles (Cq) and tested each clinical sample in duplicate. We assessed inhibition with a positive exogenous internal control (Universal Inhibition Control Cy5; Diagenode, https://www.diagenode.com). We confirmed all samples with positive qPCR results using microscopy with specific staining of microsporidia spores by the Uvitex 2B brightener (Ciba-Geigy, https://www.novartis.com) according to van Gool’s method (*22*) (Appendix Figure 1). 

### Study Population and Biologic Samples

In this study, we included all patients from center 1 who were found to be positive for *E. bieneusi* detection during January 2011–December 2018; they were considered a priori to be epidemiologically unrelated to the 3 cluster cases (M01-05 to M01-07). We included 8 supplementary control cases in the study: 4 were provided by a second university hospital, center 2 (Clermont-Ferrand, France; latitude 45.759549, longitude 3.089723), and the remaining 4 by another university hospital, center 3 (Nantes, France; latitude 47.2121974, longitude −1.554346).

### Genotyping of Clinical *E. bieneusi* Strains

#### Genotype Identification by Sequence Typing of ITS rDNA Region

We amplified the DNA extract in a 25-μL final volume and sequenced it for the ITS rDNA region. For patients with multiple positive samples, we tested only the first sample by genotyping. We used the MSP3 and MSP4B primers (*23*). ITS PCR products were purified and nucleotide sequencing performed on both strands by Eurofins Laboratories (https://www.eurofins.com). We compared the 243-bp sequences obtained with all *E. bieneusi* sequences available from the National Center for Biotechnology Information using the BLAST algorithm (http://blast.ncbi.nlm.nih.gov/Blast.cgi). We performed the evolutionary analysis of *E. bieneusi* ITS genotypes with MEGA version 7.0.26 (https://www.megasoftware.net) (*24*) and aligned all genotypes with 40 *E. bieneusi* ITS genotype sequences from GenBank(*9*).

#### Minisatellite and Microsatellite Polymorphism Genotyping

We studied the genetic diversity of the clinical strains of *Enterocytozoon bieneusi* further by analyzing 1 minisatellite (MS4) and 3 microsatellites (MS1, MS3, and MS7), as previously described (*16*). We defined sequence types by comparing MS sequences. We then performed multilocus analysis by combining ITS sequences with the 4 MS markers to define MLGs. We used the Clustal Omega algorithm (https://www.ebi.ac.uk/Tools/msa/clustalo) for sequence alignment and examined the relationship between MLGs by a median-joining network analysis using Network version 5.0.1.1 and Network Publisher version 2.1.1.2 software (http://www.fluxus-engineering.com). To confirm the clustering of cases, we also performed an evolutionary analysis of combined nucleotide sequences by the maximum likelihood method based on the Tamura 3-parameter model in MEGA version 7.0.26. We uploaded all sequences to GenBank (accession numbers in Appendix Table).

### Transmission Map

We studied patient movements within center 1 and to identify possible sites where the 3 cluster cases with *E. bieneusi* microsporidiosis (M01-05 to M01-07) may have come into contact. To do so, we extracted dates of outpatient visits and hospitalizations retrospectively from the medical records (Dossier Patient Partagé, Cerner SAS, Paris-La Défense, France).

## Results

### Description of Cluster Cases

**Figure 1:**
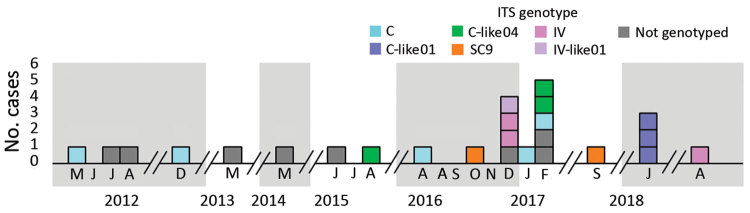
Incident number and ITS rDNA region genotypes of *Enterocytozoon bieneusi* infection cases (n = 25) in center 1 university hospital, France, January 1, 2011–December 31, 2018. ITS, internal transcribed spacer.

During January 1–December 31, 2018, we found 44 fecal samples obtained from 25 patients at center 1 positive for *E. bieneusi* (overall prevalence 0.53% of fecal samples; Figure 1; Appendix Figure 1). The cohort of microsporidiosis cases included HIV-infected persons; solid-organ transplant recipients; and patients with hematologic malignancies, autoimmune diseases, or cirrhosis (Appendix Table). The yearly frequency of diagnosis of *E. bieneusi* infection varied randomly during the study period (Figure 1). The 3 cluster cases of *E. bieneusi* microsporidiosis (M01-05 to M01-07) were diagnosed in the same hematology department over a 4-day period, January 12–16, 2018. All 3 patients had been admitted to the hospital for symptoms of diarrhea, deterioration of their general health status, or both. No individual risk behaviors, including swimming in wild rivers, consumption of nonpotable water, or close contact with animals, were identified in these patients. They resided in towns that were geographically located at distances 28.7–104.0 km apart. 

**Figure 2:**
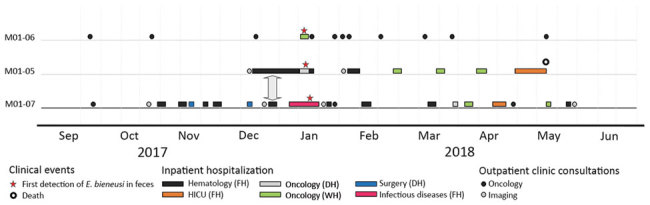
Hospitalization mapping for 3 patients with concomitant *Enterocytozoon bieneusi* microsporidiosis in the hematology unit of Center 1 university hospital, France. On the *x-*axis, dates (month-year) range from 5 months before to 5 months after the outbreak; on the *y-*axis, anonymous patient codes are given. The vertical arrow indicates the period December 26–28, 2017, when 2 patients, M01-05 and M01-07, were concomitantly housed in the same clinical department (FH in the hematology unit). DH, day hospitalization; FH, full hospitalization; HICU, hematology intensive care unit; WH, week hospitalization.

Full details of all their medical consultations and hospital admissions were available and in-hospital movements collected (Figure 2). Only 1 period for possible direct patient-to-patient contact was determined: December 26–28, 2017, in the hematology inpatient department. The patients were placed in neighboring rooms 15 and 17 days before the first sample was found positive for *E. bieneusi*, the only period of concomitant hospitalization. However, during January 11–12, 2018, patients M01-06 and M01-07 spent time in 3 different but closely located hospital wards: in the short-stay oncology unit (M01-06) and in the hematology inpatient department and the oncology outpatient unit (M01-07). These 3 clinical units are in the same hospital building on the same floor and share the same food and water supplies and some common healthcare staff.

### ITS Genotyping of Clinical *E. bieneusi* Strains

We included 33 cases in our study; 25 from center 1, 4 from center 2, and 4 from center 3 were successfully genotyped. The remaining 8 cases were unavailable for genotyping (Figure 1). On the basis of ITS rDNA sequencing, we determined that the 3 *E. bieneusi* strains from cluster cases belonged to a new genotype, closely related to genotype C (referred to hereafter as C-like01; Appendix Figure 2). We identified 2 other new genotypes in the Center 1 cohort: C-like04, which was closely related to genotype C (synonym genotype II), and IV-like01, closely related to genotype IV (synonym genotype K) (Appendix Figure 2). C-like01 and C-like04 genotypes differed from genotype C by 1 single-nucleotide polymorphism (SNP), at positions 83 (G→A) for the C-like01 genotype and 236 (G→A) for the C-like04 genotype. The IV-like01 genotype also differed from the IV genotype by 1 SNP (G→A) at position 236 (Appendix Figure 3). Other genotypes identified in our study (Appendix Table) belonged to genotypes C (n = 13), IV (n = 3), and S9 (n = 2).

### MLG Analyses 

**Figure 3:**
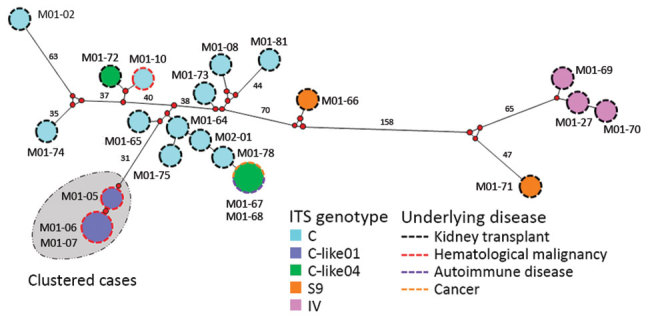
Median-joining analysis of the multilocus sequence typing (MLST) data for 22 *Enterocytozoon bieneusi* isolates from 3 different hospital centers in France, determined by using Network version 5.0.1.1 and Network Publisher version 2.1.1.2 software (http://www.fluxus-engineering.com). Circles are proportional to the frequency of each genotype (a total of 20 multilocus genotypes were obtained based on segregating sites). Pairwise differences >25 single-nucleotide polymorphisms (SNPs) are shown close to branches, which were shortened for better presentation. Gray shading indicates isolates from the cluster investigated in this study. ITS, internal transcribed spacer.

We performed MLG analyses using the combination of ITS with MS1, MS3, MS4, and MS7 loci. In 3 of the 25 isolates (M01-18, M01-77, and M01-79), >1 MS markers could not be successfully amplified (Appendix Table). Amplification efficiency was 96.0% for MS1, 92.0% for MS3 and MS7, and 88.0% for MS4. Sequence analysis of each MS marker showed a high diversity of sequence types (STs). The MS3 marker was the least variable marker, with only 5 different STs, followed by MS7 (8 STs) and MS4 (12 STs); MS1 was the most polymorphic, with 16 different STs (Appendix Table). Combination of ITS with MS markers for the 22 complete isolates (isolates for which all MS markers were successfully sequenced) resulted in 20 different MLGs (Appendix Table). Network analysis confirmed that the 3 isolates from the cluster cases in the hematology unit were very similar (Figure 3). M01-06 and M01-07 were 100% identical among the 1,904 nt positions analyzed. M01-05 differed slightly from MS7 at 1 tandem repeat. Overall, phylogenetic analyses confirmed the close relationship among the 3 isolates (Appendix Figure 4).

The MLG results, together with the short period between the occurrence of the 3 cluster cases and the association of the new C-like01 genotype with the cluster cases, provided evidence that the isolates were epidemiologically related. In addition, the MLG analyses showed a high heterogeneity among isolates belonging to the same ITS genotype. Only M01-06/M01-07 (C-like01 ITS genotype) from cluster cases and M01-67/M01-68 (C-like04 ITS genotype) were 100% identical to each other. M01-67 and M01-68 were isolated from 2 patients in Center 1 during a 7-day period in 2017. M01-67 was isolated from a 61-year-old woman admitted to the department of internal medicine with rheumatoid purpura, and M01-68 was isolated from a 70-year-old woman with hepatocellular carcinoma who died after a hospital stay of 15 days in the hepatology department. The C-like04 genotype had also been identified 2 years previously in a patient (M01-72) from center 1. However, this isolate was clearly different from the others (Figure 3).

## Discussion

The infection rate of intestinal microsporidiosis remains elevated in children in developing countries (*1*). The incidence among the HIV-positive population has been greatly reduced as a result of highly active antiretroviral tritherapy and subsequent immunity restoration (*1*). In addition to sporadic cases in travelers, infections in new populations of immunocompromised patients exposed to the disease are now emerging; these populations include solid-organ transplant recipients and patients who have cancer and hematologic malignancies. In our study, 1 of 25 patients from center 1 was HIV positive, and 15 of 25 were solid-organ transplant recipients (*20*,*25*). A recent monocentric study in a hospital in France estimated the overall prevalence of microsporidia spores in fecal samples over a 2-year period at 1.1% (*26*).

To study the epidemiology of pathogens and specifically to address outbreaks (*27*), sequence typing has long been considered to be the standard for most infectious diseases (*11*–*14*). Many previous studies focused on *E. bieneusi* were restricted to an overall determination of its genotype and investigated only nucleotide sequences of the ITS rDNA region (*28*,*29*). In 2011, Feng et al. reported a MLST-based method for *E. bieneusi* genotyping (*16*). This powerful tool has been widely used in animals and humans to decipher genetic diversity within the *E. bieneusi* species (*10*). We applied this method to an outbreak investigation. The MLST method generated a great length of microsporidian DNA containing highly variable regions ranging from 1,856 to 2,160 bp. Thus, the discriminatory power between *E. bieneusi* strains was much stronger by this technique than with ITS genotyping alone.

Using the MLST genotyping method, we defined the *E. bieneusi* strains isolated from the 3 patients admitted to the hematology unit as a new genotype (C-like01); these strains were closely clustered. This finding supported the conclusion that these patients shared the same strain, likely from a common source of contamination that remains undetermined (*18*). In our study, hospitalization mapping of the patients’ movements supported the hypothesis of nosocomial acquisition. Because this was a retrospective investigation, we were unable to evaluate the safety of the food and beverages that were provided to the patients in the hospital during the weeks before the report of the outbreak (*30*).

Reports of community-acquired foodborne or waterborne microsporidiosis outbreaks are rare. The recent literature includes the massive epidemic in Lyon, France, with an attack rate of 1.0% in HIV-positive patients each month during the summer of 1995 (*31*). At that time, molecular tools were not available to investigate the epidemiologic links between microsporidian strains. In 2009, a probable outbreak in a hotel in Sweden involving 135 persons with an attack rate of 30.0% was associated with cucumber consumption (*32*). In that study, the authors stated that 6 *E. bieneusi* strains were identified as genotype C, but no detailed genotyping was performed to investigate the genetic relationships among the isolates. The authors reported the incubation period of *E. bieneusi* infection in this outbreak ranged from 0 to 21 days.

In our study, 2 of the 3 cluster case patients were hospitalized >15 days before their first symptoms, which is compatible with nosocomial acquisition. The remaining patient was admitted to the short-stay oncology unit for 2 days when microsporidiosis occurred, but he also visited the hospital every month for regular medical consultations. We cannot exclude the possibility that the transmission of *E. bieneusi* involved asymptomatic persons, such as transiently colonized nurses and doctors or other patients as direct person-to-person contacts (*33*). However, few studies have been able to provide molecular evidence that colonized persons can serve as direct infectious sources (*3*,*12*). Comprehensive demonstration of transmission is hampered (*34*) because the time lapse for spore excretion in the feces and the minimal infective inoculum are unknown. Indirect transmission is also a possibility because the spores have protective walls formed of proteins and chitin that would allow them to persist in the environment for at least several weeks (*3*).

With respect to these findings, it is difficult to define a robust method to prevent *E. bieneusi* infection in patients at risk. Recent data support the inclusion of microsporidia on the National Institutes of Health/Centers for Disease Control and Prevention biodefense category B list of pathogens of concern for waterborne and foodborne transmission. Unfortunately, official guidelines are scarce, especially regarding persons with hematologic malignancies. In HIV-positive patients with T-cell lymphocyte CD4+ count <200 cells/μL, the Centers for Disease Control and Prevention strongly recommends reducing environmental exposure by avoiding untreated water sources (level of evidence: AIII) (*35*). Regardless of the context, we suggest that standard hygiene precautions be used, including decontamination of the hands with an alcohol-based rub. In addition, wearing personal protective equipment when in close contact with infected patients should be standard practice, as should thorough cleaning of environmental surfaces.

In conclusion, our study presents a report of 3 cluster cases of microsporidiosis in a hospital and provides strong epidemiologic and molecular evidence of a common source of contamination. We also demonstrate the high genetic diversity of *Enterocytozoon bieneusi.* Our findings suggest that the MLG approach will further extend our knowledge about the epidemiology of microsporidiosis and that incidence of nosocomial contamination may be more common than previously recognized.

Appendix Additional information about the study of *Enterocytozoon bieneusi*, France, 2012–2018.
