# Supplementary material for: Genotyping Approach for Potential Common Source of Enterocytozoon bieneusi Infection in Hematology Unit
Source: Emerg Infect Dis. 2019 Sep;25(9):1625–31. doi: 10.3201/eid2509.190311 (PMC6711236; doi:10.3201/eid2509.190311)
Supplement: Appendix — Additional information about the study of Enterocytozoon bieneusi, France, 2012–2018. [file 19-0311-Techapp-s1.pdf]

Article DOI: https://doi.org/10.3201/eid2509.190311

Genotyping Approach for Potential Common Source of *Enterocytozoon bieneusi* Infection in Hematology Unit

Appendix

Appendix Table. Characteristics of the 33 cases of *Enterocytozoon bieneusi* infection included in study, France, 2012–2018*

Strain no.	Patient age/ sex	Date of diagnosis	Underlying condition	Multilocus genotype						GenBank accession nos.
				ITS genotype	MS1 ST	MS3 ST	MS4 ST	MS7 ST	MLGs	
Center 1										
M01-73	4/F	2012 May 15	Kidney transplant	C	Type XII	Type I	Type III	Type V	MLG 14	AF101199, MK534468, MK534492, MK534474, MK534488
ND	55/M	2012 Jul 17	Cirrhosis	ND	ND	ND	ND	ND	ND	ND
ND	7/F	2012 Aug 20	Liver transplant	ND	ND	ND	ND	ND	ND	ND
M01-74	76/M	2012 Dec 14	Kidney transplant	C	Type XIII	Type I	Type XI	Type V	MLG 15	AF101199, MK534469, MK534492, MK534481, MK534488
ND	28/M	2013 Mar 6	Kidney transplant	ND	ND	ND	ND	ND	ND	ND
ND	75/F	2014 May 14	Kidney transplant	ND	ND	ND	ND	ND	ND	ND
ND	63/F	2015 Jun 16	Kidney transplant	ND	ND	ND	ND	ND	ND	ND
M01-72	64/F	2015 Aug 10	Kidney transplant	C-like04†	Type XII	Type I	Type X	Type V	MLG 13	MK531995, MK534468, MK534492, MK534480, MK534488
M01-75	62/F	2016 Jul 10	Heart transplant	C	Type XIV	Type I	Type V	Type V	MLG 16	AF101199, MK534470, MK534492, MK534476, MK534488
M01-71	75/M	2016 Oct 3	Kidney transplant	S9	Type VIII	Type III	Type IX	Type VII	MLG 12	FJ439685, MK534464, MK534494, MK534479, MK534491
ND	73/M	2016 Dec 2	Kidney transplant	ND	ND	ND	ND	ND	ND	ND
M01-70	69/M	2016 Dec 8	Kidney transplant	IV	Type XI	Type V	Type VIII	Type VIII	MLG 11	AF242478, MK534467, MK534496, MK534483, MK534490
M01-77	68/M	2016 Dec 12	HIV	IV-like01†	Type XV	NA	NA	NA	NA	MK531996, MK534471
M01-69	77/F	2016 Dec 24	Kidney transplant	IV	Type X	Type IV	Type VIII	Type VIII	MLG 10	AF242478, MK534466, MK534495, MK534483, MK534490

Strain no.	Patient age/ sex	Date of diagnosis	Underlying condition	Multilocus genotype						GenBank accession nos.
				ITS genotype	MS1 ST	MS3 ST	MS4 ST	MS7 ST	MLGs	
M01-78	67/F	2017 Jan 13	Kidney transplant	C	Type IX	Type I	Type II	Type VI	MLG 17	AF101199, MK534465, MK534492, MK534473, MK534489
M01-68	69/F	2017 Feb 2	Hepatocellular carcinoma	C-like04†	Type IX	Type I	Type II	Type VI	MLG 9	MK531995, MK534465, MK534492, MK534473, MK534489
M01-79	65/M	2017 Feb 6	Inflammatory bowel disease	C	NA	NA	NA	NA	NA	AF101199
M01-67	61/F	2017 Feb 9	Rheumatoid purpura	C-like04†	Type IX	Type I	Type II	Type VI	MLG 9	MK531995, MK534465, MK534492, MK534473, MK534489
ND	53/M	2017 Feb 11	Myeloma	ND	ND	ND	ND	ND	ND	ND
ND	78/F	2017 Feb 24	Lymphoma	ND	ND	ND	ND	ND	ND	ND
M01-66	71/F	2017 Sep 26	Kidney transplant	S9	Type VIII	Type III	Type VII	Type VII	MLG 8	FJ439685, MK534464, MK534494, MK534478, MK534491
M01-06‡	59/M	2018 Jan 12	Chronic Myeloid Leukenia	C-like01†	Type II	Type I	Type II	Type III	MLG 3	MK531994, MK534457, MK534492, MK534473, MK534486
M01-05‡	57/M	2018 Jan 13	Lymphoma	C-like01†	Type II	Type I	Type II	Type II	MLG 2	MK531994, MK534457, MK534492, MK534473, MK534485
M01-07‡	63/M	2018 Jan 16	Lymphoma	C-like01†	Type II	Type I	Type II	Type III	MLG 3	MK531994, MK534457, MK534492, MK534473, MK534486
M01-27	67/M	2018 Aug 21	Kidney transplant	IV	Type XVI	Type V	Type VIII	Type VIII	MLG 20	AF242478, MK534461, MK534496, MK534483, MK534490
Center 2										
M01-18	32/F	2010 Mar 11	Immuno-comptetent	C	Type V	Type I	NA	Type VI	NA	AF101199, MK534460, MK534492, MK534489
M01-10	10/M	2014 May 12	Acute myeloid leukemia	C	Type IV	Type I	Type IV	Type V	MLG 5	AF101199, MK534459, MK534492, MK534475, MK534488
M01-08	73/M	2017 Jan 26	Kidney transplant	C	Type III	Type I	Type III	Type IV	MLG 4	AF101199, MK534458, MK534492, MK534474, MK534487
M01-02	72/F	2018 Feb 15	Kidney transplant	C	Type I	Type I	Type I	Type I	MLG 1	AF101199, MK534456, MK534492, MK534472, MK534484
Center 3										

Strain no.	Patient age/ sex	Date of diagnosis	Underlying condition	Multilocus genotype						GenBank accession nos.
				ITS genotype	MS1 ST	MS3 ST	MS4 ST	MS7 ST	MLGs	
M01-81	59/F	2013 Jan 22	Kidney transplant	C	Type VI	Type I	Type XII	Type II	MLG 18	AF101199, MK534462, MK534492, MK534482, MK534485
M02-01	5/F	2013 Aug 29	Kidney transplant	C	Type VI	Type I	Type II	Type V	MLG 19	AF101199, MK534462, MK534492, MK534473, MK534488
M01-65	59/F	2014 Mar 5	Kidney transplant	C	Type VII	Type II	Type VI	Type V	MLG 7	AF101199, MK534463, MK534493, MK534477, MK534488
M01-64	56/F	2015 Jul 10	Kidney/ pancreas transplant	C	Type VI	Type I	Type V	Type V	MLG 6	AF101199, MK534462, MK534492, MK534476, MK534488

*NA, not amplified; ND, no DNA; ITS, internal transcribed spacer; MLG, multilocus genotype; MS, microsatellite; ST, sequence type.
† New genotype.
‡ Cluster cases.

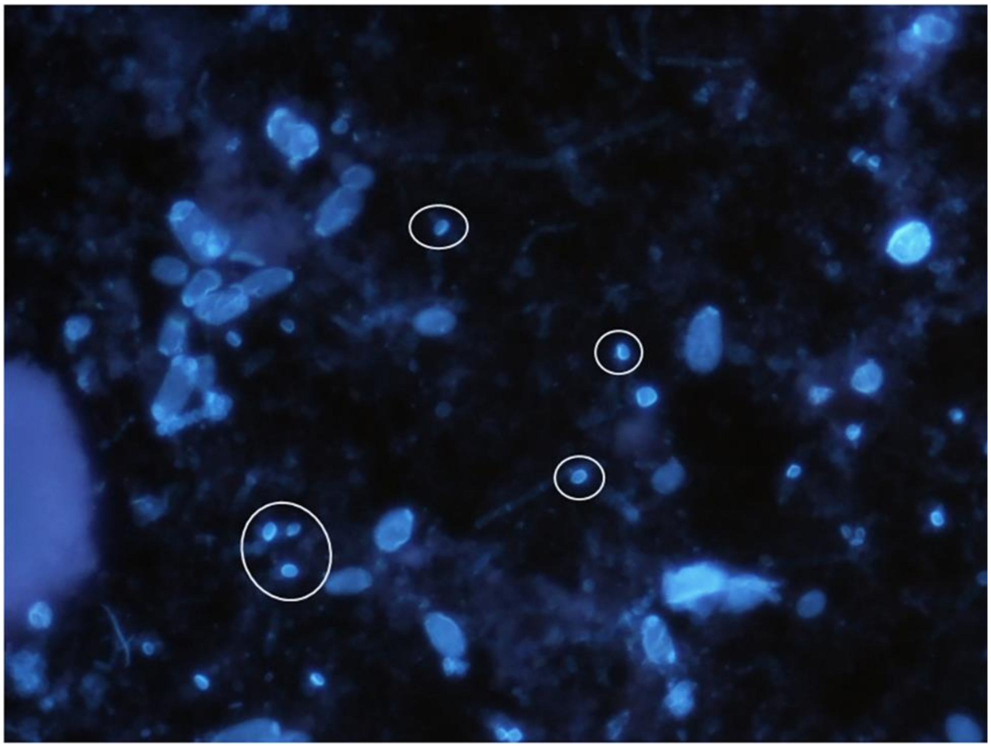

Appendix Figure 1. Microscopic observation of a fecal sample containing *Enterocytozoon bieneusi* spores (Uvitex 2B fluorescent brightener, https://www.polysciences.com; original magnification 1,250×). Spores are encircled. They are round-to-oval shaped, small sized (2–5 μm long), and contain a filament that crosses the endoplasm.

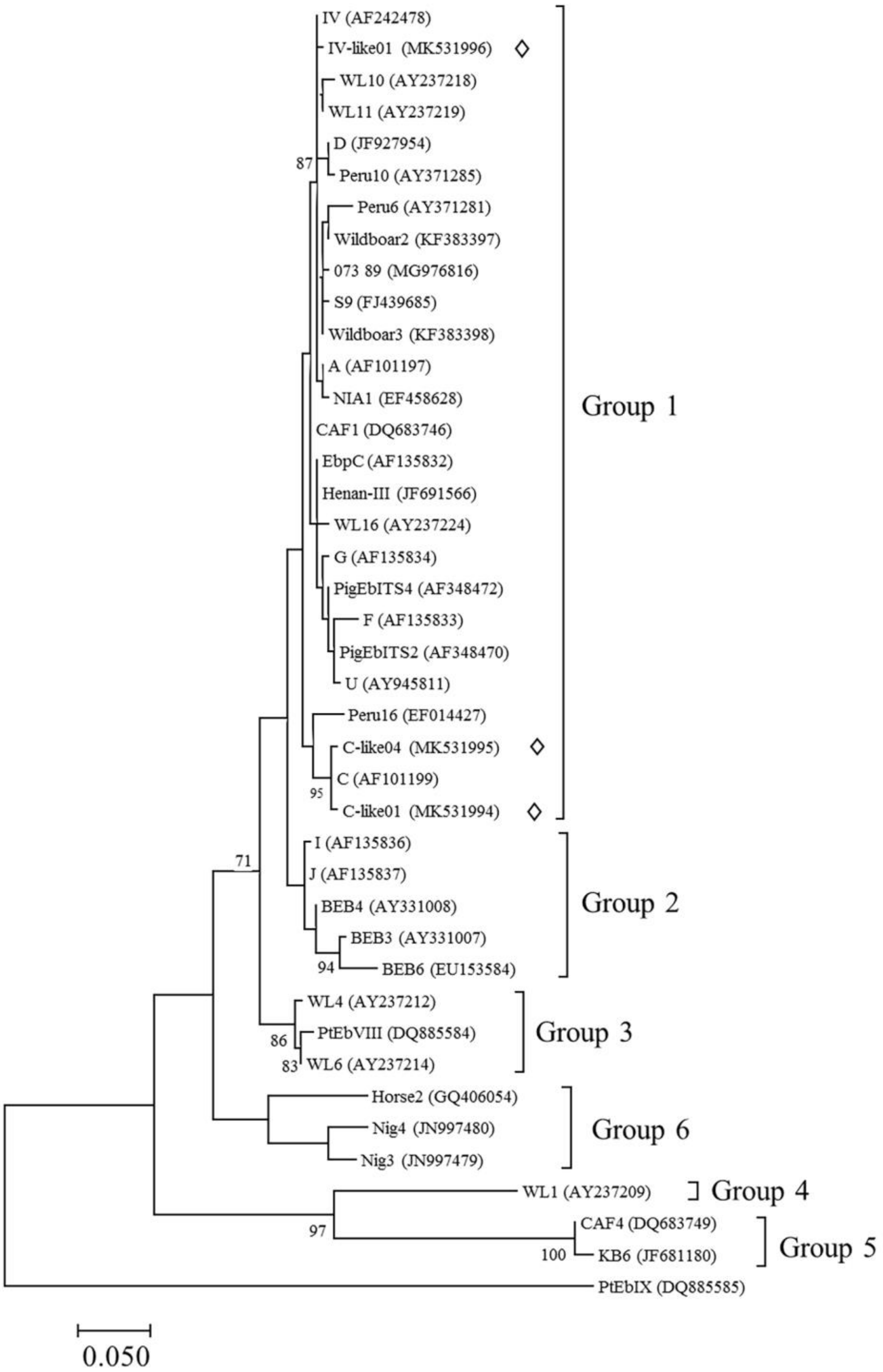

Appendix Figure 2. Molecular phylogenetic analysis of ITS rDNA genotypes for the *Enterocytozoon bieneusi* strains by the maximum likelihood method. Maximum likelihood phylogenetic tree (MEGA7 v7.0.26 software, https://www.megasoftware.net) based on 42 ITS nucleotide sequences from *Enterocytozoon bieneusi*, including new genotypes. Kimura 2-parameter model was used with 10,000 bootstrap replications. Bootstrap values >70% are shown. PtEbIX strain was used as an outgroup. ◊ denotes genotype(s) newly identified in the present study.

```
                1        10        20        30        40        50        60
                |         |         |         |         |         |         |
C               TCAGTTTTTGGGGTGTTGGTATCGGAATGTATGGTAGGTGATGTGTGTGTGTATGGGGGA
C-LIKE01        TCAGTTTTTGGGGTGTTGGTATCGGAATGTATGGTAGGTGATGTGTGTGTGTATGGGGGA
C-LIKE04        TCAGTTTTTGGGGTGTTGGTATCGGAATGTATGGTAGGTGATGTGTGTGTGTATGGGGGA

C               TGCCGAGGGGACCAGCGGTGTGGTGGTGTGTGTAGGCGTGAGAGTGTATCTGTAAGGGTG
C-LIKE01        TGCCGAGGGGACCAGCGGTGTGATGGTGTGTGTAGGCGTGAGAGTGTATCTGTAAGGGTG
C-LIKE04        TGCCGAGGGGACCAGCGGTGTGGTGGTGTGTGTAGGCGTGAGAGTGTATCTGTAAGGGTG

C               AGGAATGTGGGTGCAGCGAGTTAGAGGTTGTTCCATGTGGAATAGTGGGATTGGTACATG
C-LIKE01        AGGAATGTGGGTGCAGCGAGTTAGAGGTTGTTCCATGTGGAATAGTGGGATTGGTACATG
C-LIKE04        AGGAATGTGGGTGCAGCGAGTTAGAGGTTGTTCCATGTGGAATAGTGGGATTGGTACATG

C               ATGGTTGGATGGGGGAATGATGTGTGTATGGGTGAGGAAAATCGGAGGTTGCGGTGCGAG
C-LIKE01        ATGGTTGGATGGGGGAATGATGTGTGTATGGGTGAGGAAAATCGGAGGTTGCGGTGCGAG
C-LIKE04        ATGGTTGGATGGGGGAATGATGTGTGTATGGGTGAGGAAAATCGGAGGTTGCGGTACGAG

C               CGG
C-LIKE01        CGG
C-LIKE04        CGG


                1        10        20        30        40        50        60
                |         |         |         |         |         |         |
IV              TCAGTTTTTGGGGTGTGGGTATCGGAATGTGTGGTAGGTGATGTGTGTGTGTATGGGGGA
IV-like01       TCAGTTTTTGGGGTGTGGGTATCGGAATGTGTGGTAGGTGATGTGTGTGTGTATGGGGGA

IV              TGCCGAGGGGACCAGCGGTGCGGTGGTGTGTGTAGGCGTGAGAGTGTATCTGCAAGGGTG
IV-like01       TGCCGAGGGGACCAGCGGTGCGGTGGTGTGTGTAGGCGTGAGAGTGTATCTGCAAGGGTG

IV              AGGGATGTGGGTGCAGCGAGTTAGAGGTGGTTCCATGTGGAATAGTGGGATTGGTACGTG
IV-like01       AGGGATGTGGGTGCAGCGAGTTAGAGGTGGTTCCATGTGGAATAGTGGGATTGGTACGTG

IV              ATGGTTGGATGGGGGAATGATGTGTGTATGGGTGAGGAAAATCGGAGGTTGCGGTGCGAG
IV-like01       ATGGTTGGATGGGGGAATGATGTGTGTATGGGTGAGGAAAATCGGAGGTTGCGGTACGAG

IV              CGG
IV-like01       CGG
```

Appendix Figure 3. Multiple alignment of new genotypes identified in the study.

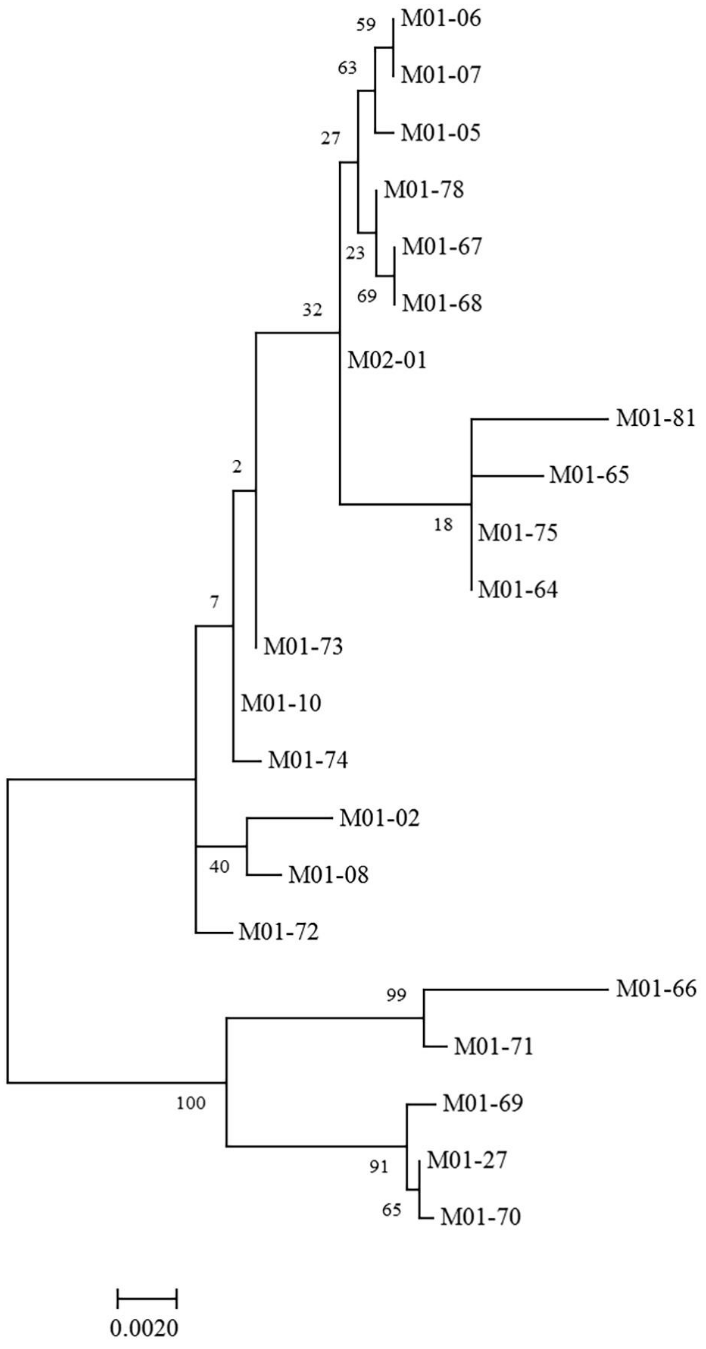

Appendix Figure 4. Molecular phylogenetic analysis of concatenated sequences (ITS rDNA, MS1, MS3, MS4, and MS7) genotypes for the *Enterocytozoon bieneusi* strains by the maximum likelihood method. Maximum likelihood phylogenetic tree (MEGA7 v7.0.26 software, https://www.megasoftware.net) based on 22 MLG nucleotide sequences from *Enterocytozoon bieneusi* using Tamura 3-parameter model with a discrete gamma distribution (10,000 bootstrap replications).
